# Pathways to resilient rural livelihoods: Lessons from Southwestern Uganda

**DOI:** 10.4102/jamba.v17i1.1905

**Published:** 2025-09-20

**Authors:** Betty C. Mubangizi

**Affiliations:** 1 College of Law and Management Studies, School of Management, IT and Governance, University of KwaZulu-Natal, Durban, South Africa

**Keywords:** rural livelihoods, pandemics and disasters, resilience, social networks, vulnerability, SW Uganda

## Abstract

Disasters and pandemics have significantly disrupted livelihoods in rural regions of southwestern Uganda. However, there remains limited understanding of the resilience processes and recovery pathways available to these communities. This study addresses that gap by applying the three capacities resilience framework, comprising absorptive, adaptive and transformative capacities, to investigate how rural communities in Isingiro and Kisoro districts respond to and recover from such shocks. Using a qualitative methodology that included 34 key informant interviews and four focus group discussions, the research explores the vulnerabilities faced by rural populations, the role of institutions and the strategies employed at household and community levels to withstand and adapt to disruptions. Findings reveal that while state and non-state actors provide critical support, much remains focused on absorptive responses, offering short-term relief without fostering sustainable change. In contrast, adaptive strategies, such as reliance on indigenous knowledge and social networks, offer more community-driven resilience. The study also highlights the need for building transformative capacity, which remains underdeveloped but is essential for long-term livelihood security and systemic resilience.

**Contribution:**

The findings underscore the need for long-term planning that integrates institutional frameworks with community-based approaches to enhance resilience. To build transformative capacity, substantial investments in addressing economic vulnerabilities, fostering income diversification and strengthening community participation in governance are critical. This study situates its conclusions within the resilience framework, emphasising that sustainable recovery requires collaborative efforts across institutional and community levels to build systems capable of absorbing shocks, adapting to changes and transforming in the face of future challenges.

## Introduction

Rural areas face multiple challenges in various parts of the world. The challenges stem from, among others, disasters and pandemics. Floods, earthquakes, landslides and droughts are disasters many emerging economies often have to grapple with (Chingono & Mbohwa 2023). These economies also face a range of communicable and non-communicable diseases. The former, which are generally preventable and treatable, include emerging and re-emerging infectious diseases, which are perhaps the most intimidating threats to the human race and responsible for a considerable burden of morbidity and mortality (Mustafa & Makhawi 2021). Ebola, Marburg virus disease, human immunodeficiency virus (HIV) or acquired immunodeficiency syndrome (AIDS) and coronavirus disease 2019 (COVID-19) are some of the epidemic communicable diseases, of which the latter two have become pandemics. While these diseases last, they adversely impact the livelihoods of the affected rural communities. Education, income and overall well-being are adversely affected.

Uganda has had difficult experiences of disasters, particularly floods, in the recent and far past. These events, intricately related to climate change, have caused significant disruptions of livelihoods across communities. Because of climate-related events, displacement and sometimes resettlement of persons are rife in affected areas. Uganda’s migration, environment and climate change nexus has been studied and reported (Twinomuhangi et al. 2022). The study focused on displacements in three areas: flood- and drought-induced migrations in Katakwi district (Teso sub-region), landslide-induced migrations in Bududa district (Mt Elgon sub-region) and drought-induced migrations in Amudat (Karamoja sub-region). In each area, communities were highly vulnerable to disasters and had little, if anything, that they could do to stop the threats posed by the disasters. Temporary out-migration can be an affordable way to escape disaster, and migration can thus be a coping mechanism for environmental and climate stresses. Temporary out-migration emerged as a practical coping strategy, allowing affected individuals to relocate, either seasonally or episodically, to safer areas where they could access livelihoods, shelter or social support. The Karamoja sub-region has reported seasonal migrations during drought and prolonged dry periods. Drought deprives pastoralists of green pastures for their cattle, compelling them to move towards Napak and Katakwi districts in search of water and pasture (Twinomuhangi et al. 2022).

The dynamics of disastrous floods during the COVID-19 pandemic have been analysed in Uganda’s Kasese District (Bamutaze 2020). The study used the Sendai Framework for Disaster Risk Reduction (to which Uganda is a signatory) to assess the implementation measures to realise the key tenets of the framework and promote a disaster-resilient society. Findings indicated that although Uganda is prone to disasters because of its physical environment and the vulnerability of its population, the number of hazards experienced between February and May 2020 was unprecedented. These stretched the ecological and social systems to their limits. These included the worst locust infestation in 70 years, record floods and landslides, all occurring alongside the COVID-19 pandemic. The joint effects of floods and COVID-19 were particularly severe in the study district. One of the conclusions was that future recovery and reconstruction efforts should preferably consider the co-occurrence of natural hazards with biohazards (such as COVID-19 or Ebola) to enhance disaster resilience. There was also a need to encourage the participation of the private sector throughout the disaster response. The Kasese case underscores the importance of viewing disasters not as isolated events but as complex, compounding crises that test the absorptive, adaptive and transformative capacities of affected communities – core dimensions of resilience as defined by global frameworks such as the Sendai Framework and Intergovernmental Panel on Climate Change (IPCC) reports. These dynamics necessitate a closer examination of how institutions and mobility strategies like temporary migration interact to shape community-level responses to vulnerability and cumulative risk.

In the context of this study, a disaster is defined as a sudden or prolonged event, whether natural, biological, or socio-political, that significantly disrupts livelihoods, strains community resources and overwhelms local coping mechanisms (UNDRR 2015). This includes floods, droughts, pandemics like COVID-19 and cross-border conflicts compromising rural household security and well-being. In this study, we distinguish between *disasters*, which refer primarily to climate- and environment-induced events such as floods, landslides and droughts, and *pandemics*, which are health-related biological crises such as COVID-19. While both shocks disrupt rural livelihoods, they differ in their causes, manifestations and institutional response requirements.

### Problem statement

While rural communities in Uganda are disproportionately affected by disasters and pandemics, current resilience strategies remain largely fragmented, short-term and insufficiently informed by these contexts’ institutional and socio-cultural realities. This study critically examines how formal and informal governance systems interact with community-level coping mechanisms to influence the resilience trajectory of rural households, highlighting a gap in transformative approaches to long-term livelihood security. Despite global emphasis on resilience, there remains a critical gap in understanding how resilience is built and sustained in these contexts, particularly in relation to institutional and governance systems. This study addresses this gap by examining how formal and informal institutions influence the capacity of rural households to absorb, adapt to and recover from intersecting crises such as floods and pandemics. It focuses on marginalised rural districts where recurring environmental shocks have been compounded by the COVID-19 pandemic, exploring how governance arrangements, local coping mechanisms and temporary migrations shape recovery trajectories and long-term resilience.

### Objectives of the study

This study assessed the resilience and sustainability of rural livelihoods in the Isingiro and Kisoro districts of Southwestern Uganda. The following specific objectives guided it:

- To investigate the impact of disasters and pandemics on rural livelihoods from a local governance perspective.
- To identify the vulnerabilities of rural livelihoods and the institutional factors that either increase the rural communities’ susceptibility or strengthen their resilience.
- To explore the strategies adopted by rural communities to diversify income sources and enhance the long-term sustainability of their livelihoods.

### Conceptual framework

**FIGURE 1:**
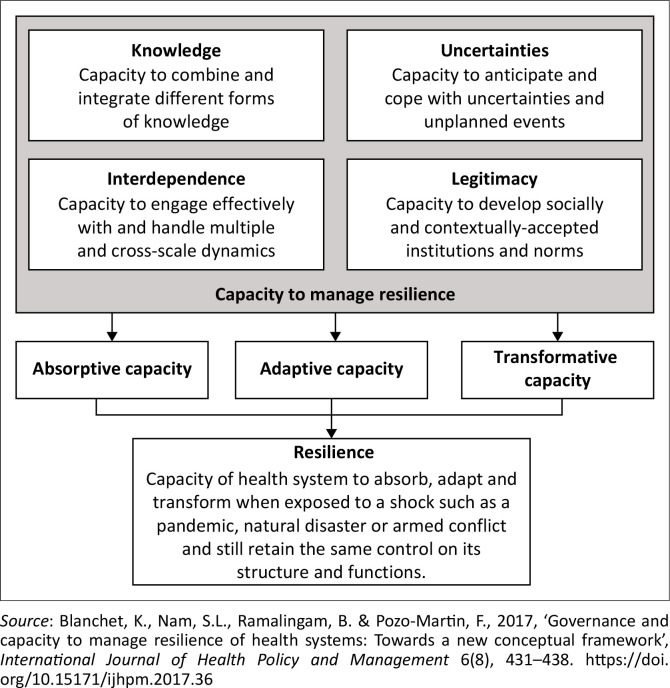
A conceptual framework of resilience governance.

The concept of resilience has been widely adopted across various disciplines, including public health (see, for example, Kieny et al. 2014). Today, there is a broad consensus that the global community has to help build more resilient health systems. This is because strengthening the capacity of health systems to manage resilience is critical to continue delivering essential healthcare services to populations effectively. Some researchers see the strength of a health system as its capacity to absorb, adapt and transform when exposed to a shock such as a pandemic, disaster or armed conflict, while still retaining the same control over its structure and functions (Blanchet et al. 2017). Health systems can exhibit resilience at three levels: absorptive capacity, adaptive capacity and transformative capacity (Figure 1). By adapting Blanchet et al.’s health systems resilience framework to the broader domain of rural livelihoods, the study offers a tailored conceptual model that links structural (institutional, community and household) drivers with proximate factors (absorptive, adaptive and transformative capacities) in shaping resilience outcomes.

Absorptive capacity refers to a health system’s ability to continue delivering essential healthcare services and maintaining population protection during a shock by relying primarily on existing resources, infrastructure and routines (Blanchet et al. 2017; Kruk et al. 2015). Adaptive capacity involves the ability of the system to adjust its operations and reconfigure processes to maintain service delivery levels in the face of disruption, often through reallocation of resources or changes in service delivery models (Hanefeld et al. 2018; Kruk et al. 2017). Transformative capacity, meanwhile, denotes the ability of a health system to fundamentally alter its structure, functions, or governance in response to long-term contextual changes or repeated shocks, thereby increasing its sustainability and responsiveness over time (Blanchet et al. 2017; Thomas et al. 2020).

**FIGURE 2:**
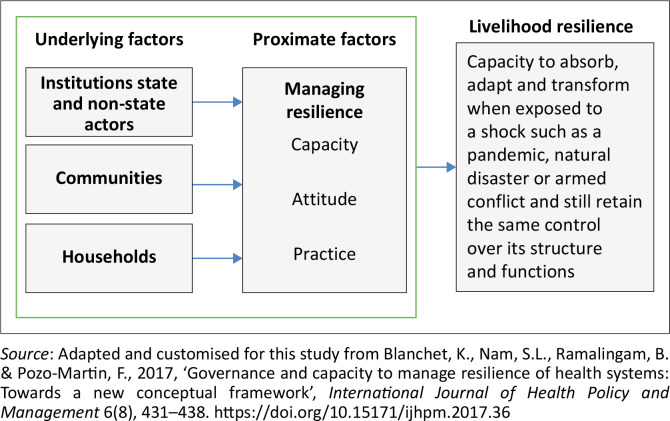
Conceptual framework underpinning livelihood resilience.

The framework used in this discussion is partly informed by the health system resilience framework articulated previously. However, our framework focuses on broader livelihood resilience rather than health system resilience. Further, although we find the framework invaluable, we cannot use all its components and thus select a few concepts to inform our study. Consequently, we hypothesise that background factors (institutional, community and household factors) operate through intermediate factors (capacity, attitude and practice) to influence livelihood resilience. Livelihood resilience is the capacity of a system to absorb, adapt and transform when exposed to a shock while still retaining control over its structure and functions (Blanche et al. 2017; Mubangizi 2024; Scoones 1998). Thus, a livelihood system is resilient if it exhibits absorptive, adaptive or transformational capacity in the face of shocks of different intensities (Figure 2).

Figure 1 and Figure 2 present complementary perspectives on resilience. While Figure 1 (adapted from Blanchet et al. 2017) originates from the health systems field and elaborates the internal capacities, such as knowledge, legitimacy and interdependence, needed to manage resilience, our adapted framework in Figure 2 recontextualises these insights for the analysis of livelihood systems. Specifically, Figure 2 simplifies and reorganises the broader conceptual terrain by focusing on how underlying contextual factors (institutions, communities and households) interact with proximate drivers (capacity, attitude and practice) to shape resilience outcomes in rural livelihoods. Together, these frameworks inform a layered understanding of resilience. Figure 1 offers a granular view of what resilience entails conceptually. In contrast, Figure 2 translates these concepts into a functional model for analysing community-based and livelihood-oriented resilience processes in marginalised rural settings.

### Study area background

**FIGURE 3:**
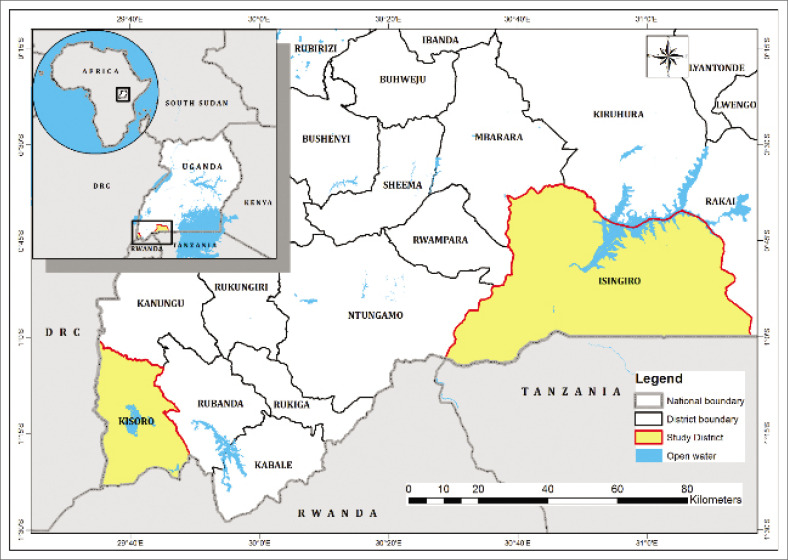
Study districts, Uganda.

The study was conducted in Isingiro and Kisoro districts, located in the southwestern region of Uganda (Figure 3). These districts are part of Uganda’s highland zones. They are characterised by diverse topography, including steep slopes and valleys that make them highly susceptible to natural hazards such as floods, landslides and soil erosion, dangers that have increased in frequency and intensity because of climate variability and unsustainable land use practices.

Isingiro District, which shares a border with Tanzania, has historically faced challenges related to cross-border migration, livestock movement and water scarcity while also serving as a key transit route for both goods and people. Kabuyanda sub-county is primarily agrarian, marked by smallholder farming, limited road infrastructure and inadequate access to health and social services, factors that compound vulnerability during environmental shocks.

Kisoro District, lying near the borders of Rwanda and the Democratic Republic of Congo, is similarly mountainous and remote, with Nyakabande sub-county being both geographically isolated and densely populated. The district frequently experiences landslides triggered by heavy rainfall, which displace households and disrupt livelihoods. The area’s remoteness, fragmented administrative reach and under-resourced public infrastructure heighten local institutions’ governance and disaster response challenges.

These contextual characteristics – geographical isolation, socio-economic marginalisation and exposure to recurrent shocks – make Isingiro and Kisoro districts important for examining livelihood resilience, particularly how institutional and household-level dynamics interact to shape recovery and adaptation in rural settings. Areas with low exposure to such hazards or with robust institutional support systems were excluded to maintain focus on highly at-risk rural settings.

### Sampling strategy and data collection

The study employed purposive sampling to select both the geographical areas and the participants. The districts of Isingiro and Kisoro were chosen based on their high vulnerability to disasters, history of flood and landslide events, and rural character, which align with the study’s focus on resilience in marginalised communities. Within these districts, Kabuyanda and Nyakabande sub-counties were selected as representative sites because of their exposure to multiple hazards and limited infrastructure. For participant selection, key informants (KIs) and focus group participants were identified based on their knowledge, roles and experience in local governance, disaster response and rural service delivery. This approach ensured the inclusion of diverse and relevant perspectives on institutional responses and community-level resilience practices.

This study used a qualitative research approach to examine the impact of disasters and pandemics while exploring the role of local governments in supporting rural livelihoods and sustainability. Fieldwork utilised KI interviews and focus group discussion guides, prepared in advance and translated into local languages for accessibility. The variables in the conceptual framework, such as institutional factors, community practices and household capacities, were directly operationalised through the design of the Key Informant Interviews (KII) and Focus Group Discussions (FGD) guides. These tools were structured to probe themes such as institutional responsiveness, livelihood strategies, attitudes towards risk and resilience practices, thereby aligning the data collection process with the conceptual categories guiding the analysis.

The data collection team included district coordinators, field supervisors and sub-county research assistants. Researchers conducted 36 one-on-one interviews with KIs selected for their expertise and involvement in local governance, rural development and service provision. These informants included local government officials such as Sub-County Councillors, Parish Executive Committee Chairs, Chief Administrative Officers (CAOs), Local Council V (LCV) Chairpersons, District Planners (DPs), District Environment Officers (DEnvOs) and District Health Officers (DHOs). Representatives from nongovernmental organisations (NGOs) engaged in health-related work also participated.

Four focus group discussions involved local men and women actively engaged in socio-economic activities within their sub-counties, providing diverse perspectives on community resilience and governance.

**FIGURE 4:**
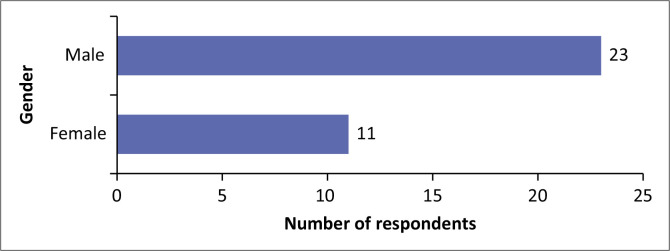
Gender of the respondents.

Figure 4 shows that male KIs (23, or 67.6%) significantly outnumbered female informants (11, or 32.4%). This disparity reflects the predominance of men in local government leadership positions. The finding aligns with reports from the Economic Policy Research Centre and UN Women, highlighting Uganda’s ongoing challenges in achieving gender parity in political representation at the local government level (UN Women 2022).

A total of four focus group discussions were conducted in the districts. Gender balance was considered, leading to one male and one female group discussion per district. Six people participated in each of the group discussions. Participants were purposively selected to reflect diverse socio-economic and livelihood backgrounds, including small-scale farmers, informal traders, youth representatives and elderly community members. Facilitators were also trained to create a safe and respectful environment where all voices, especially those of women and other potentially marginalised participants, could be heard and valued. The focus group discussions were organised to capture a broader range of community perspectives, engaging participants from various socio-economic backgrounds, including residents and stakeholders actively involved in rural livelihood activities. These discussions allowed for in-depth dialogue about the lived experiences of rural community members and the challenges they face in accessing services and engaging in livelihood strategies.

The study employed thematic interviews and focus group data analysis, using Dedoose software for systematic coding and theme identification on local government, service provision and rural development. The relevant institutions granted ethical clearance.

### Ethical considerations

Ethical clearance to conduct this study was obtained from University of KwaZulu-Natal Humanities and Social Sciences Research Ethics Committee (No. HSSREC/00004560/2022), Makerere University College of Humanities and Social Sciences School of Social Sciences Research Ethics Committee (No. MAKSSREC 10.2024.717) and the Uganda National Council for Science and Technology (No. SS2544ES).

## Results

The study investigates how recent global events, particularly disasters and pandemics, have affected rural livelihoods and focuses on the role of local governance and institutional determinants in shaping resilience and sustainability. It provides an overview of disasters and pandemics that have recently impacted the southwestern Uganda districts of Isingiro and Kisoro. The analysis highlights how institutional frameworks have either exacerbated vulnerabilities or contributed to the resilience of rural livelihoods. Attention is also given to the strategies employed by rural communities to enhance the long-term viability of their livelihoods. In addition, the study examines the roles played by social networks and community-based organisations in supporting community resilience and adaptive capacities.

### The nature of disasters and pandemics in the study area

**FIGURE 5:**
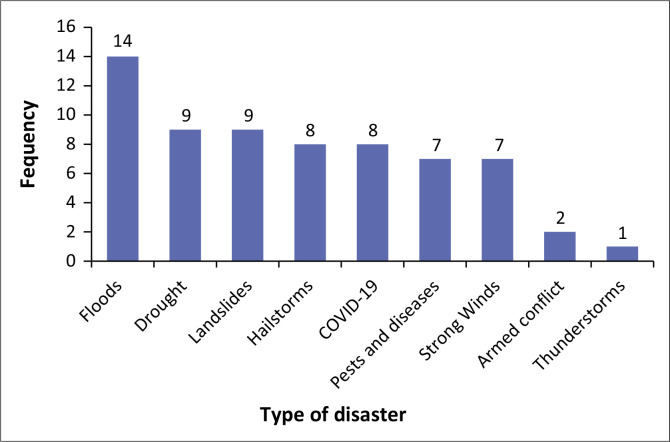
Reported frequency of disasters.

Figure 5 shows the frequency of disasters and pandemics reported in the study areas over the last 5 years. As the figure indicates, the most mentioned disaster was floods, as several participants explained:

‘Floods from Kanywamaizi in Kaguto-Ruhenda. This water swept away crops, and the place where this water gathered formed a lake named Kigaaga. These floods caused hunger, and people are still facing this challenge. This happened 2 years back.’ (KII, adult, male)

‘We experience *imyuzure* [*floods*] and much water running downslope. In the event of such occurrences, famine sets in. We have seen this in Nyakabande village, especially in the Ruborogota area. When floods occur, diseases almost always follow. Floods and rains occur almost on an annual basis.’ (FGD, female, small scale farmer)

The severity of drought and heavy winds was also reported, as one participant explained:

‘Here, we get a long drought, where we do not have any rain, which has critically affected Kabuyanda’s agriculture. For those who do farming, their businesses get destroyed in the event of a severe drought.’ (KII, adult, male)

Other disasters mentioned were landslides, hailstorms and COVID-19. Thunderstorms, pests and diseases were further pointed out. Participants noted a range of pests and diseases affecting humans and animals. One participant, for example, had this to say:

‘The pests and diseases include “*Muwowa*” [*blight*], which affects tomato plants. When the disease strikes, all tomatoes become red even before maturity. There is foot and mouth disease as well as chicken pox that affects our animals every year. Then there is Kwashiorkor among children. Malnutrition also leads to a lack of food because the crops dry up during drought. Hence, children lack a balanced diet for proper growth.’ (FGD, adult, female)

Armed conflict was identified as a disaster, causing significant suffering in communities. The conflict between M23 militants and government forces in the Democratic Republic of Congo displaced people and led to a refugee influx in the Kisoro district. This conflict severely disrupted rural livelihoods, further straining local resources and resilience.

Drought, landslides, strong winds and floods which damaged crops were reported to have undermined food security and the incomes of rural communities. Coronavirus disease 2019 further exacerbated their vulnerability by causing widespread loss of life and forcing the closure of most economic activities.

### The influence of disasters and pandemics on livelihoods

Participants highlighted the diverse impacts of disasters and pandemics on rural livelihoods. Crops and foodstuffs were among the most affected, with beans, potatoes and banana gardens partially or completely destroyed in the hilly areas of Nyakabande sub-county. Drought in the Kabuyanda sub-county reduced water harvesting and caused widespread water shortages in homes. Floods disrupted education by preventing learners from travelling to school, and some students dropped out entirely as household poverty worsened.

Participants in a focus group discussion vividly described the profound impact of COVID-19 on livelihoods, emphasising its widespread effects on communities. One of them had this to say:

‘People were usually not able to work. There was hunger, business ended, schools closed, and transport was disrupted. There was domestic violence. This arose due to the difficulty of the husband and wife being together at home for weeks and months when the former was not providing anything, including basics such as soap. “Husband was viewed as of no use and the shame and powerlessness of the husband would translate into anger, hence violence.”’ (KII, adult male, community leader)

‘Some people changed their drink type from the usual local sorghum brew to *Waragi* [*a local potent gin*] and took to drugs. Some perceived Waragi as a cure for the pandemic. Unfortunately, the Waragi heightened drunkenness, and the corollary of it was an elevated level of domestic violence.’ (FGD, adult, male)

‘Further, decadence and delinquency among school-going age children increased as children no longer attending school gradually turned to unethical practices.’ (FGD, adult, female)

Pandemics aside, disasters were similarly pointed out as having negatively affected rural livelihoods over the last 10 years. Several KIs had the following to say:

‘Drought reduced access to food and water. Disasters have also led to loss of lives.’ (KII, adult, male)

‘In terms of disaster, livelihoods are the most affected. For example, if a person is harvesting about 100 bunches of matooke when heavy winds come, he makes losses. On several occasions, we have had this Sub County flooding, affecting agriculture and leading to soil erosion.’ (KII, adult, male, community officer)

Although pandemics and disasters are well known for their adverse effects, an interesting finding was the revelation of the positive aspect of COVID-19, its harmful effects notwithstanding. Participants in one of the focus group discussions generally agreed that although the pandemic disrupted normal livelihoods and brought misery to individuals, households and communities, it nonetheless had its positive side. The reported positive outcomes included the urge to work harder amid challenges and enhanced morals, as one participant put it:

‘“Women became more forward-looking and hardworking. They devised means of putting food on the table instead of relying on their husbands”. Also, the uptake of zero grazing improved since moving around villages with livestock was no longer tenable. Further, engagement with crafts was boosted through practices such as mat-making and hat-making using local grass called “ishinge” [*lovegrass – Eragrostis sp.*]. The COVID-19 period was the only time for young girls to be home with their mothers and learn a little craft-making.’ (FGD, adult, male, community leader)

### Institutional framework and livelihood resilience strategies in the face of disasters and pandemics

This research explored how institutions support community livelihoods in the face of disasters and pandemics. Participants highlighted various institutions that assisted during and after calamities. Government and non-governmental organisations played crucial roles, along with international agencies like Medical Team International, Oxfam, United Nations High Commissioner for Refugees and the Red Cross (UNHCR), which operate in the study districts. These organisations provided essential food, tents, basins and tarpaulins. One participant shared:

‘Red Cross has built a latrine block for Nyakabande sub-county. It also provided food, tarpaulins, liquid soap and saucepans for those affected by landslides. FAWE has supported single mothers and helps make energy-saving stoves.’ (FGD, adult, female, community member)

These institutional interventions contribute to resilience in multiple ways. The immediate provision of food and shelter supplies strengthens the absorptive capacity of communities by enabling them to withstand shocks without resorting to harmful coping strategies. Medium-term initiatives, such as the construction of latrines, the promotion of energy-saving technologies and support for vulnerable groups like single mothers, enhance adaptive capacity by reducing exposure to future hazards, promoting sustainable practices and diversifying livelihood options. In some cases, these efforts also lay the groundwork for transformative capacity, especially where institutional actors help reshape infrastructure, foster innovation, or build local skills and governance structures that strengthen long-term preparedness and autonomy.

Thus, institutional support in the study areas is not only reactive but also contributes meaningfully to resilience-building pathways by bolstering the community’s ability to absorb, adapt and, potentially, transform in the face of recurrent shocks.

The Parish Development Model (PDM) and the Development Response to Displacement Impact Project (DRDIP) were identified as key sources of support during times of need. Development Response to Displacement Impact Project, funded by the World Bank and implemented by Uganda’s Office of the Prime Minister (OPM), assists vulnerable nationals and refugees in refugee-hosting districts. Its goals include improving access to essential social services, expanding economic opportunities and enhancing environmental management. Development Response to Displacement Impact Project operates through several components: social-economic infrastructure development, sustainable environment and natural resource management, economic empowerment and project management, monitoring and evaluation.

Respondents cited DRDIP and PDM as institutional frameworks that were supporting communities in the Kabuyanda sub-county, as one participant put it:

‘DRDIP is a helpful organisation. It sensitises women, and the government funds them to develop their businesses. TASO has also come in to help us, especially HIV/AIDS patients, through counselling and giving drugs; they also teach us how to eat healthy foods and how to take drugs in time. PDM has helped us get capital, pay debts, and improve our livelihoods and those that have not received this money have hopes of getting it because they registered in groups.’ (FGD, adult, female, healthcare, worker)

Some participants acknowledged the support provided by the Central Government of Uganda. It was pointed out that the government often rescued communities in need through the OPM. One KI had this to say:

‘When heavy winds hit us, the government provided food items to the affected people, and some schools were given iron sheets to replace those that were blown away. People were given seeds to plant in the next season; they were also given trees to plant and protect soils.’ (KII, adult, male, community, member)

Another KI added:

‘The government has tried to provide food and medicine in case of disasters and money to work on infrastructure. It also sensitises people on how they can overcome these disasters. In case of heavy rains that have damaged crops, government officials try to sensitise the community to re-plant trees and manage plantations to avoid hunger or any other problems that may come.’ (KII, adult, female, community, member)

Stakeholders indicated that the sub-county administration compiles lists of affected persons and forwards the document of victims needing support to the district administration. The district-level management, in turn, forwards the names to OPM, the institution responsible for disaster management. However, although OPM intervenes, the time lag between disaster onset and support receipt can be quite long. Office of the Prime Minister starts by mobilising requisite resources and arranging to transport relief items to the affected communities. In the event of a dangerous disaster, OPM, in conjunction with the Uganda Red Cross Society, considers modalities through which the affected persons are relocated to safer places, such as schools, as a first aid measure.

Key informants reported that the sub-county administration also enforces guidelines in case of a pandemic or disease outbreak to curb the spread. For example, during COVID-19, the sub-county administration enforced standard operating procedures (SOPs) to control the disease, which had been set by the Ministry of Health and President’s Directives. In that way, SOPs were adhered to, a practice that contributed to the control of the disease.

Key informants further reported that sub-counties sensitise communities to combat or reduce the adverse effects of disasters and pandemics. Examples cited are the sensitisation of the rural people to control flash floods and soil erosion on steep hillslopes. Sensitisation messages include practising land furrowing and replanting trees to reduce the speed of water run-off.

Responses to disasters and pandemics sometimes take the form of actions by local communities. The study examined community responses to disasters, highlighting disaster management policies, preparedness committees and community sensitisation efforts to mitigate impacts. Key informants noted practices like constructing garden gullies and land furrowing to reduce soil erosion. Alongside government and NGO support, the African concept of ‘ubuntu’ fosters solidarity, with friends and relatives providing refuge and assistance to those affected.

These institutional actions collectively shape pathways to resilience by supporting all three dimensions of resilience. The sub-county’s role in enforcement and sensitisation contributes to the community’s absorptive and adaptive capacities, enabling people to maintain basic functioning and adjust behaviours during crises. Meanwhile, the OPM and humanitarian agencies like the Red Cross contribute to transformative capacity by facilitating relocation, mobilising resources and integrating disaster risk reduction into local systems. From prevention and preparedness to recovery, these layered interventions illustrate how institutions frame and reinforce the resilience agenda, linking immediate response with long-term community adaptation and sustainability.

### Expanding incomes to improve the viability of livelihoods in the face of disasters and pandemics

One way of remaining buoyant in the face of pandemics and disasters is to have reliable and sufficient income. Unfortunately, even the little income available gets depleted when the calamities strike. Communities are then forced to find means and ways of survival. This research sought to understand whether and how rural communities were making any effort to expand their incomes to improve their long-term viability of livelihoods. Participants indicated that some efforts were being made to raise incomes through undertakings such as investment in agriculture, trading and joining SACCOs, as some participants put it:

‘People have tried to work tirelessly to improve agriculture, and also, people have engaged in trading in the business sector. They have also engaged themselves in education. When you educate children, you are developing a village because education is now the key to development. They have started SACCOs to save money and borrow at low interest to do business and pay children’s fees. They have formed groups, which has greatly helped them.’ (KII, adult, female)

‘We have institutions that skill the youth and provide employment opportunities and incentives to work. People have changed from subsistence to plantation agriculture, where they can rear animals and plant crops for sale. The water dam project is boosting this, and they will be cultivating.’ (KII, adult, male)

Respondents highlighted several programmes initiated by the Ugandan government to improve local livelihoods and enhance community resilience. These programmes aim to reduce poverty, provide financial support, empower youth, create employment opportunities and enhance welfare through income-generating projects. Key initiatives include the PDM, Youth Livelihood Programme (YLP), Uganda Women Entrepreneurship Programme (UWEP) and *Emyooga (skills)*.

The PDM, a 5-year initiative, focuses on transitioning Ugandans from subsistence farming to a money-based economy. Its primary objectives are to increase household incomes, improve the quality of life and achieve economic transformation by moving households out of subsistence farming and integrating them into the money economy within 5 years. However, participants did not report any observable or tangible changes in household incomes attributable to the PDM at the time of data collection, suggesting that its impact, if any, had not yet materialised or was not widely felt in the study areas.

Respondents highlighted additional government programmes designed to improve livelihoods, create jobs and support marginalised groups, including the *Presidential Initiative on Wealth and Job Creation (Emyooga)*, the *YLP* and the *UWEP*.

Launched in 2019, the *Emyooga* initiative transforms households from subsistence to market-oriented production. Its primary goal is to promote job creation and enhance household incomes as part of the government’s broader strategy for economic transformation. This programme targets groups engaged in various economic activities and encourages collective action for improved productivity.

The YLP, introduced in the fiscal year 2013–2014, targets unemployed and poor youth aged 18–30. Its goal is to empower these youth to harness their socio-economic potential, enabling them to create self-employment opportunities and increase their income levels. The programme explicitly supports youth who have dropped out of school or training institutions, those without formal education, single parents, individuals with disabilities, youth living with HIV/AIDS and those who have completed formal education but remain unemployed.

The UWEP aims to enhance women’s access to financial services and equip them with skills for enterprise growth, value addition and product marketing. Managed by the Ministry of Gender, Labour and Social Development, UWEP seeks to empower women economically by providing affordable credit, facilitating access to markets, promoting appropriate production technologies and building capacity for entrepreneurship.

Together, these programmes aim to address critical gaps in economic empowerment, poverty reduction and inclusive development, focusing on the unique needs of youth, women and other vulnerable groups. Some KIs acknowledged the assistive role of the programmes, as one elaborated:

‘Through government programs like the Youth Livelihood Programme, youths are expanding their income areas through Emyooga. PDM targeting the local population under 39% poverty level is helpful.’ (KII, adult, male)

Similarly, participants in focus group discussions mentioned the government’s supporting role, as one put it:

‘An upcoming irrigation project will provide us with water for irrigation. This will improve our gardens so we can have food all the time. The government has brought us banks which give us loans. Some groups got money for Emyooga and have used it for development.’ (FGD, adult, female)

These findings reflect broader arguments in the sustainable livelihoods framework (Chambers & Conway 1992; Scoones 1998), highlighting the importance of expanding access to financial, natural and institutional assets to strengthen household resilience. Such programmes contribute to both adaptive capacity, enabling households to diversify livelihoods and respond flexibly to shocks, and transformative capacity, creating opportunities for long-term structural improvements in rural well-being. As Mubangizi (2024) argues, effective institutional support that links local governance with development interventions can catalyse inclusive rural development, particularly when initiatives address structural inequities and are responsive to local realities. In this regard, programmes like the PDM and *Emyooga*, though still nascent, hold potential to influence the resilience pathways of rural communities by shifting them from subsistence dependency toward sustainable economic participation.

### Social networks and indigenous wisdom

This research sought to understand the role of friends, families, people of shared origin and communities in fortifying their livelihood resilience. It further enquired into how association networking could bolster individual, household and community efforts against pandemics and disasters. The role networks and indigenous knowledge played was explored, particularly concerning COVID-19. Findings indicate that networks were helpful because some individuals and households could share knowledge and support one another. Findings further indicate that people shared knowledge of agricultural practices much to their mutual benefit. Some people used indigenous knowledge to contribute to the management of COVID-19. Several participants explained:

‘Some people use social media such as WhatsApp, which enables others to try ways of managing diseases. However, this applied to only those who could charge their phones at home: those who ordinarily had to move to charging points outside their homes were cut out. Others used the radio to listen in to messages. Note that during COVID-19, there were no visits. A person could die, and people would learn much later that so and so died and was even buried.’ (KII, adult, youth leader)

‘People increasingly turned to the use of organic materials such as ‘Blackjack’/’Bidens Pilosa’ while others turned to leaves of eucalyptus trees in a bid to fight COVID-19. These materials were practical and helpful.’ (FGD, adult, female)

Another key livelihood strategy investigated during the study was the role of social networks like the Village Savings and Loan Association (VSLA) and Savings and Credit Cooperative Organisations (SACCOs) in helping vulnerable rural people. A VSLA is a group of people who meet regularly to save together and take small loans from those savings. The group’s activities run in cycles of 1 year, after which the accumulated savings and the loan profits are distributed back to the members. The purpose of a VSLA is to provide simple savings and loan facilities in a community that does not have easy access to formal financial services.

Savings and Credit Cooperative Organisations are financial institutions owned, managed and governed by their members. They are self-help organisations where members pool their savings and lend them to each other at agreed-upon rates and conditions. They are an important alternative for people whom traditional financial institutions may not serve. They help people develop a savings habit and investment culture and can provide loans for various needs, such as weddings, education, home renovations and health.

Village Savings and Loan Associations and SACCOs are regulated by the Uganda Microfinance Regulatory Authority (UMRA), a Government Regulatory Agency (https://umra.go.ug/). Established under the *Microfinance Institutions and Money Lenders Act*, UMRA is an autonomous Government Agency. It was formed to promote a sound non-banking financial institutions sector. The institutions include savings and credit cooperatives, village saving and loan associations, non-deposit-taking microfinance institutions and money lenders. Ultimately, UMRA works to enhance financial inclusion, financial stability and financial consumer protection among the lower-income population in Uganda (https://umra.go.ug/).

Stakeholders reported that Saving groups like (SACCOs and VSLAs) help to inculcate the saving culture in the rural people. This is done by providing platforms where the local community can save money in their locality. The saving groups also provide loans at a reasonable rate compared to moneylenders, who charge higher interest rates. Furthermore, the saving groups at the village level avoid the bureaucracy of financial institutions that require someone to have collateral security to access credit, which rural people in the target study area cannot afford. Stakeholders reported that some NGOs have played a critical role in helping the rural community enhance their resilience by skilling young people in various vocational skills and sensitising them to other livelihood initiatives. Friends and close families were reported to help settle the disaster and help the affected persons.

These community-based financial organisations contribute significantly to the resilience pathways of rural households. By facilitating access to savings and credit, VSLAs and SACCOs strengthen absorptive capacity, enabling members to manage everyday risks and buffer the immediate impacts of shocks without selling critical assets. They also support adaptive capacity by providing capital for small investments in livelihood diversification, agricultural improvement, or micro-enterprises, which enhance the ability of households to adjust to changing economic and environmental conditions. Over time, participation in these organisations can contribute to transformative capacity, fostering greater financial independence, social cohesion and the development of community-led safety nets that reduce long-term vulnerability (Béné et al. 2012; Mubangizi 2024).

## Discussion

The results of this study provide critical insights into the resilience of rural livelihoods in Southwestern Uganda, particularly in the face of disasters and pandemics. The findings reveal a complex interplay between institutional frameworks, community responses, social networks and indigenous knowledge in shaping resilience outcomes. This discussion contextualises these findings within the resilience framework, emphasising the capacities to absorb, adapt, transform and recover as influenced by state and non-state actors, communities and households.

### The impact of disasters and pandemics on rural livelihoods

Isingiro and Kisoro districts have experienced multiple episodes of disasters and pandemics in the recent and far past. The findings reveal the profound and multidimensional impacts of disasters and pandemics on rural livelihoods in Kisoro and Isingiro districts, Southwestern Uganda.

Floods, droughts and strong winds emerged as the most frequently mentioned disasters, aligning with findings from other studies in sub-Saharan Africa, highlighting the prevalence of climatic shocks in rural settings (Adger, Arnell & Tompkins 2005; Kikstra et al. 2022; Nikoloski, Christiaensen & Hill 2018). These disasters damage crops like beans, bananas and potatoes and disrupt education, access to water and infrastructure. This mirrors findings from across Africa, where floods and drought cycles have similarly exacerbated food insecurity and eroded household resilience (Reed et al. 2022).

In the Kisoro district, armed conflict from the Democratic Republic of Congo exacerbated vulnerabilities by straining local resources, echoing patterns in border regions of the Sahel, where cross-border conflicts intensify resource competition and disrupt livelihoods (Hendrix & Brinkman 2013). These findings underscore the importance of building institutional and community resilience to address cross-border impacts of conflict.

The COVID-19 pandemic presented an unprecedented shock, disrupting economic activities, education and social networks. It had significant adverse effects, including loss of life, income disruption and increased household poverty. However, the finding of a negative and positive impact is notable. Participants cited enhanced morals and a renewed work ethic as unexpected outcomes of the pandemic. This duality aligns with studies in Kenya and South Africa documenting pandemic-induced innovation in income diversification and heightened community solidarity (Makoni 2020; Okem et al. 2023).

The absorptive capacity of rural households, characterised by their reliance on local resources and traditional coping mechanisms, was severely tested during the pandemic. For example, the closure of schools and economic activities mirrors findings from other African contexts where education and informal economies were disproportionately affected (UNICEF 2021). However, the adaptive responses observed, such as working harder and fostering social cohesion, demonstrate an intrinsic resilience rooted in indigenous knowledge and community support systems.

These absorptive and adaptive responses reflect crucial stages in the rural resilience pathway. Absorptive capacity, which involves drawing on local resources and traditional practices, enables rural households to endure shocks in the short term without undergoing fundamental change, preserving core livelihood functions despite adversity. Adaptive capacity, on the other hand, reflects the ability of communities to learn from and adjust to stressors, often by modifying livelihood strategies, strengthening informal social safety nets, or integrating new practices based on past experiences. Together, these capacities form the building blocks of a resilience pathway that allows communities not only to recover from crises such as pandemics but also to strengthen their systems against future disruptions incrementally. In this way, local and communal solidarity act as enablers of sustained adaptation and long-term resilience (Béné et al. 2012; Mubangizi 2024).

### Institutional framework and livelihood resilience strategies

The role of institutional frameworks in enhancing community resilience against disasters and pandemics is pivotal in rural Uganda, as demonstrated in this study. At various levels, local, national and international institutions play distinct but complementary roles in supporting rural livelihoods, offering critical insights into absorptive, adaptive and transformative capacities as outlined in the resilience framework.

### Institutional contributions to absorptive capacity

Providing immediate relief items such as food, tents and other essentials by organisations like Medical Team International, Oxfam, UNHCR and the Red Cross reflects the critical absorptive function of institutions. These interventions mitigate the immediate impacts of disasters, preventing the total collapse of livelihood systems. Similar patterns have been observed in Mozambique, where international agencies provided relief after Cyclone Idai to stabilise affected communities (Emerton et al. 2020).

The government’s intervention through the OPM and district-level coordination also underscores institutional absorptive capacity. However, the noted delays in mobilising and delivering resources reveal a critical gap. Delayed responses reduce the effectiveness of absorptive capacity, which is highly time-sensitive. Comparable findings from Kenya indicate that reducing logistical delays in relief distribution enhances community resilience (Mwangi & Anaya 2020). This finding aligns with the theoretical framing of resilience as comprising time-sensitive, multi-scalar responses that involve both institutional and community-level mechanisms (Folke et al. 2010). From an institutional theory perspective, the effectiveness of absorptive capacity is not only a function of resource availability but also of the institutional arrangements and processes that govern decision-making, coordination and responsiveness (Peters 2019). Delays in response highlight institutional inertia or procedural rigidity, which can undermine the intended buffering function of state-led disaster interventions. Thus, enhancing institutional absorptive capacity requires not just the presence of emergency support structures but also the agility to activate them quickly in high-risk contexts – especially where communities rely on state institutions as their primary line of defence during crises.

### Strengthening adaptive capacity through development projects

Longer-term initiatives like the PDM and the DRDIP demonstrate how institutions contribute to adaptive capacity. Development Response to Displacement Impact Project’s integrated approach enhances social services, economic opportunities, environmental management and the multidimensional nature of resilience. By improving infrastructure, promoting economic empowerment and addressing environmental challenges, such projects help communities adapt to ongoing and future shocks.

These findings resonate with studies from Rwanda, where development-focused projects enhanced agricultural productivity and income diversification, bolstering community resilience (World Bank, 2023). Uganda’s focus on empowering host communities and refugees through DRDIP is particularly noteworthy, as it prevents resource competition and promotes coexistence, a common challenge in refugee-hosting African districts.

### Transformative capacity and long-term institutional support

Transformative resilience involves structural and functional shifts to better prepare for future challenges. While the findings highlighted immediate relief efforts and adaptive strategies, this study also reveals steps toward transformation, such as community sensitisation to manage environmental hazards. Sub-county administrations’ efforts to encourage tree planting, land furrowing and flood management showcase transformative initiatives.

However, these initiatives remain constrained by institutional inefficiencies and resource limitations. Comparable experiences in Ethiopia demonstrate that transformative capacity is significantly enhanced when local governments integrate traditional knowledge systems with modern practices, ensuring that resilience efforts are contextually relevant and sustainable (Teshome et al. 2019).

### Institutional challenges and gaps

While institutions play a crucial role in enhancing community resilience, the research findings highlight several challenges that hinder their effectiveness. Respondents emphasised issues such as delays in disaster response and the limited capacity of institutions to enforce disaster management guidelines. These limitations undermine the timeliness and effectiveness of interventions, leaving affected communities more vulnerable to the impacts of disasters and pandemics.

Addressing these challenges requires strengthening coordination mechanisms between local and central governments. Faster and more streamlined communication processes could significantly reduce delays in mobilising and delivering disaster response resources, as evidenced by studies on disaster governance in Malawi, which illustrate how improved coordination enhances the effectiveness of institutional frameworks (Chikodzi & Nhamo 2021). Enhancing community involvement in institutional decision-making could bridge responsiveness and inclusivity gaps. Participatory approaches, where community insights and needs are integrated into institutional planning, have improved resilience outcomes, as demonstrated in resilience-building initiatives in Tanzania (Mwakatobe et al. 2020). By addressing these challenges, Uganda’s institutional frameworks could better support communities in mitigating and recovering from the impacts of disasters and pandemics.

### Governance during pandemics

The enforcement of SOPs during COVID-19 exemplifies the role of governance in mitigating the spread of pandemics. The enforcement of SOPs by sub-county administrations reflects the institutional absorptive capacity to maintain health standards and protect livelihoods during a crisis. Studies from South Africa also emphasise the critical role of governance in pandemic control, highlighting that timely and consistent communication builds public trust and compliance (Mokoena et al. 2022).

A recurring theme in the findings is the complementary roles of local and international institutions. International agencies provide essential resources and technical expertise, while local institutions enforce policies, educate communities and oversee implementation. This synergy is consistent with findings from Nigeria, where collaborative efforts between NGOs and local governments significantly improved disaster response and recovery (Abaje et al. 2019).

### Expanding incomes to improve livelihood viability

The findings highlight the critical role of income diversification and economic empowerment initiatives in enhancing the resilience of rural livelihoods to disasters and pandemics. These efforts align with the transformative capacity framework, emphasising structural shifts that reduce vulnerability and foster long-term sustainability. Income diversification is essential for communities to remain buoyant in the face of shocks. Participants emphasised investments in agriculture and trade and participation in SACCOs. These efforts reflect the adaptive capacities of rural communities, enabling them to cushion the impacts of shocks and maintain economic stability. Similar patterns have been observed in Nigeria and Kenya, where SACCOs and small-scale farming have proven effective in enhancing household resilience (Adeola & Evans 2017; Ogalo & Rugami, 2024).

Savings and Credit Cooperative Organisations, in particular, play a dual role in resilience-building: providing access to financial resources and fostering social capital. The collective savings model allows members to access affordable credit, which is often critical during recovery. This aligns with findings in Rwanda, where SACCOs have been instrumental in funding income-generating activities for smallholder farmers (Ndoruhirwe, Mugenzi & Karangwa 2024).

### Government-led initiatives for economic empowerment

The study underscores the significant contributions of government programmes, including the PDM, YLP, UWEP and *Emyooga*, in improving livelihoods. These programmes exemplify transformative resilience by addressing systemic challenges such as poverty, unemployment and gender inequality:

- *Parish Development Model*: By transitioning households from subsistence to commercial farming, the PDM promotes economic transformation and long-term sustainability. This mirrors Rwanda’s Girinka programme, which integrated vulnerable households into market systems through livestock-based livelihoods, yielding substantial income gains and food security improvements (Munyaneza et al. 2020).
- *Youth Livelihood Programme:* Targeting unemployed and marginalised youth, YLP empowers participants to create self-employment opportunities. Similar youth-focused interventions in South Africa and Ghana have successfully reduced poverty and promoted social inclusion through skill development and microfinance support (Boateng, 2021; Mokoena & Phago, 2020).
- *Uganda Women Entrepreneurship Programme:* UWEP addresses gender-specific barriers by providing women with financial services and entrepreneurial skills. Such initiatives resonate with Kenya’s Women Enterprise Fund, which has enhanced women’s economic participation through capacity-building and access to affordable credit (Kinyanjui & Ocholla 2024).
- *Emyooga Initiative:* By promoting collective action for market-oriented production, the Emyooga initiative aligns with transformative resilience frameworks. Encouraging cooperative efforts among economic groups enhances productivity and reduces vulnerability, a proven effective strategy in Ethiopia’s Productive Safety Net Programme (PSNP) (Gilligan et al. 2014).

### Challenges in income expansion efforts

Despite the commendable efforts through initiatives like the PDM and Emyooga, significant challenges continue to hinder the expansion of income-generating efforts in rural communities. One of the primary obstacles is the low availability of resources. Limited access to essential inputs such as land, agricultural supplies and credit restricts the ability of households to transition effectively into market-oriented production, thereby undermining the objectives of these programmes.

Another critical challenge is the lack of market accessibility. Many rural communities struggle to connect their products to viable markets, significantly reducing the profitability of their income-generating activities. This disconnect prevents rural producers from fully capitalising on their efforts and limits the potential economic impact of their enterprises.

Additionally, awareness gaps persist, with many community members either unaware of these government programmes or unable to access them equitably. This lack of information and unequal access create disparities in the reach and impact of the initiatives, leaving vulnerable populations underserved.

### The role of social networks and indigenous wisdom in livelihood resilience

The study highlights the critical contributions of social networks and indigenous wisdom in enhancing livelihood resilience among rural communities in Uganda. These elements bolster absorptive and adaptive capacities by fostering mutual support, promoting knowledge sharing and facilitating access to financial resources. Below is a detailed discussion framed within the resilience framework and supported by comparative insights from the African context.

### Social networks and community resilience

Social networks, including families, friends and people of shared origin, play a vital role in fortifying individual, household and community resilience. These networks provide emotional, material and informational support during crises. As reported in the study, sharing agricultural knowledge and best practices exemplifies adaptive resilience, allowing communities to address immediate challenges while building capacity for future shocks.

The concept of Ubuntu, emphasising interconnectedness and collective well-being, is central to African social structures and reflects the relational dynamics observed in the study. Similar findings in Kenya and South Africa highlight how informal networks provide food, shelter and financial assistance during disasters (Chikanda & Crush 2017; Moekena & Phago, 2020; Mubangizi et al. 2023). These networks serve as informal safety nets, especially in contexts where formal institutions may be absent or inadequate.

Savings groups like VSLAs and SACCOs emerged as critical mechanisms for enhancing financial inclusion and supporting resilience. By offering accessible credit and fostering a culture of saving, these organisations empower rural communities to recover from disasters and pandemics.

Village Savings and Loan Associations are grassroots savings groups that provide flexible financial services to members, allowing them to pool resources and access small loans. By avoiding the high interest rates and collateral requirements of traditional financial institutions, VSLAs support the absorptive capacity of vulnerable households. Similar initiatives in Ghana and Tanzania have demonstrated that VSLAs enhance economic stability and improve the capacity of households to withstand shocks (Allen & Staehle 2021; Mwakatobe et al. 2020).

Savings and Credit Cooperative Organisations are regulated by the UMRA, providing a formalised yet accessible financial model for rural communities. They bridge the gap between informal savings groups and formal financial institutions, fostering economic empowerment. Studies in Rwanda Ndoruhirwe et al. 2024 similarly emphasise SACCOs’ role in enhancing livelihood diversification and reducing dependency on subsistence agriculture (Ndoruhirwe, Mugenzi & Karangwa 2024).

### Challenges and gaps

While valuable, social networks and indigenous knowledge face notable challenges and limitations. Informal networks often lack the resources to address large-scale crises, necessitating complementary support from formal institutions and NGOs. Additionally, access to these networks is not always equitable, with vulnerable groups such as widows, persons with disabilities and the elderly frequently excluded. Furthermore, Indigenous knowledge is not consistently recognised or integrated into formal policy frameworks, undermining its potential impact. Addressing these gaps requires intentional efforts to foster collaboration between local networks, formal institutions and development agencies.

### Conclusion and pathways to strengthening resilience

In this study, the resilience pathway is conceptualised as a progressive process in which communities first rely on absorptive capacity (coping with immediate shocks using existing resources), then develop adaptive capacity (adjusting strategies to reduce future risk) and ultimately aim for transformative capacity (systemic change that restructures institutions and livelihoods). This sequence is not strictly linear but reflects an evolving capacity-building continuum influenced by institutional support, social networks and local agency. The pathway guides the data analysis and the interpretation of how rural communities transition from survival to long-term resilience.

The findings of this study underscore the interconnected roles of state and non-state institutions, communities and households in fostering the capacity to absorb, adapt, transform and recover from the impacts of disasters and pandemics. The resilience framework, focusing on absorptive, adaptive and transformative capacities, provides a robust lens to understand how these actors interact to mitigate vulnerabilities and enhance livelihood sustainability in Southwestern Uganda. State institutions, such as the OPM, provide emergency relief and implement long-term development programmes like the PDM and the DRDIP. Complementing these efforts, non-governmental organisations and community-led initiatives, such as VSLAs and SACCOs, act as critical conduits for fostering financial inclusion, knowledge-sharing and social support. In synthesising these findings, the study draws on resilience theory as conceptualised by scholars such as Folke et al. (2010) and Béné et al. (2012), who emphasise that resilience is not a static condition but a dynamic process involving absorptive, adaptive and transformative capacities. The resilience pathway observed in this study moves from short-term coping (absorptive capacity), through medium-term adjustments (adaptive capacity), toward long-term systemic change (transformative capacity). This progression is mediated by endogenous factors – such as social capital, indigenous knowledge and community-based institutions – and exogenous interventions from state and non-state actors. Thus, strengthening resilience in rural Uganda requires a multi-level, multi-actor strategy that enhances community agency while reforming institutional responsiveness and resource governance. Integrating resilience thinking into policy and practice ensures that recovery efforts restore the status quo and enable communities to better withstand and transform in the face of future shocks.

Rural communities’ adaptive and transformative capacities are particularly evident in their ability to utilise social networks, indigenous knowledge and local governance structures to manage risks and recover from crises. Practices such as land furrowing, tree planting and leveraging cultural frameworks like *Ubuntu* demonstrate the strength of local solutions in addressing shocks. However, the effectiveness of these strategies is significantly enhanced when integrated with institutional support and external interventions. This aligns with the observations of Mubangizi and Adekanla (2024), who highlight that resilience to disasters is not just a function of institutional capability but also hinges on the capacity of households and communities to adapt and transform in response to systemic vulnerabilities.

Ultimately, the study confirms that resilience is a dynamic process shaped by institutional governance, community solidarity and household resourcefulness. For Uganda’s rural livelihoods, the path to recovery and sustainability lies in strengthening this synergy. Drawing on the insights of Mubangizi and Adekanla (2024), who emphasise the critical need for inclusivity and participatory governance in resilience-building, this study advocates for policies and programmes holistically integrating state, non-state and community efforts. By fostering collaboration across these levels, Uganda can build a more robust and equitable framework to address the multifaceted challenges of disasters and pandemics.

Theoretically, these findings reinforce the central tenets of resilience theory by demonstrating how absorptive, adaptive and transformative capacities manifest in rural livelihood systems under pressure. Institutional delays and short-term relief efforts reflect limited absorptive capacity, while community innovations and diversified income strategies illustrate adaptive responses. However, the limited evidence of systemic change highlights a gap in transformative capacity. This underscores the theoretical insight that resilience is not merely about bouncing back but evolving forward through structural reform. The study thus extends resilience theory by showing how context-specific governance and social capital shape each stage of the resilience pathway.

The findings emphasise the need to strengthen resilience at multiple levels of absorptive, adaptive and transformative capacities to mitigate the negative impacts of disasters and pandemics effectively. The following pathways provide actionable strategies for enhancing resilience across communities, households and institutions.

### Enhancing absorptive capacity

Strengthening absorptive capacity involves ensuring that communities can maintain essential functions during crises. Key measures include improving market access by building infrastructure that links rural producers to markets, thereby increasing the profitability of their income-generating activities. Expanding financial inclusion is another critical pathway; scaling up SACCOs and microfinance institutions can provide vulnerable households with affordable credit to weather economic shocks. Additionally, integrating Indigenous knowledge into formal disaster preparedness policies can ensure culturally relevant and resource-sensitive approaches to managing crises, such as using traditional agricultural practices and local health remedies.

### Building adaptive capacity

Adaptive capacity focuses on enabling communities to adjust to changing circumstances and reduce long-term vulnerabilities. Enhancing the functionality and reach of SACCOs and VSLAs is essential to foster financial stability and flexibility. Promoting inclusive programmes that ensure equitable access to government initiatives, particularly for marginalised groups such as women, youth and persons with disabilities, is vital for fostering equity. Moreover, integrating capacity-building efforts, including entrepreneurial skills training, financial management and sustainable livelihood practices, can improve the effectiveness of programmes like the UWEP and the YLP.

### Advancing transformative capacity

Transformative capacity entails fostering systemic changes that address root causes of vulnerability and enable long-term resilience. Supporting initiatives that promote vocational training and skill development can help diversify income sources and empower communities to transition from subsistence to market-oriented production. Strengthening grassroots resilience through targeted training and resources for local leaders and community groups can create a foundation for sustained transformation. NGOs and external support also play a critical role by addressing structural inequalities, raising awareness about sustainable livelihood practices and fostering local ownership of development projects.

### Leveraging social networks and community engagement

Social networks, including SACCOs, VSLAs and informal support systems, provide critical safety nets during crises. Institutional support for these networks should be expanded, ensuring equitable access and enhanced financial management capabilities. Inclusive community engagement is equally important, with targeted programmes that involve marginalised groups in resilience-building initiatives. Recognising and formalising the role of indigenous knowledge within policy frameworks can further enhance the relevance and efficacy of resilience strategies.

### Addressing market and resource challenges

Overcoming market and resource challenges is essential for maximising the impact of resilience initiatives. Improved programme implementation, equitable resource allocation and infrastructure development are critical for supporting market access and resource distribution. These efforts are crucial for realising the full potential of income expansion strategies and fostering inclusive economic growth in rural communities.
